# l-2-Haloacid dehalogenase (DehL) from *Rhizobium* sp. RC1

**DOI:** 10.1186/s40064-016-2328-9

**Published:** 2016-05-20

**Authors:** Aliyu Adamu, Roswanira Abdul Wahab, Fahrul Huyop

**Affiliations:** Department of Biotechnology and Medical Engineering, Faculty of Biosciences and Medical Engineering, Universiti Teknologi Malaysia, 81310 Johor Baharu, Johor Malaysia; Department of Chemistry, Faculty of Science, Universiti Teknologi Malaysia, 81310 Johor Baharu, Johor Malaysia

**Keywords:** DehL, *Rhizobium* sp. RC1, Dehalogenation, Catalytic amino acid residues

## Abstract

l-2-Haloacid dehalogenase (DehL) from *Rhizobium* sp. RC1 is a stereospecific enzyme that acts exclusively on l-isomers of 2-chloropropionate and dichloroacetate. The amino acid sequence of this enzyme is substantially different from those of other l-specific dehalogenases produced by other organisms. DehL has not been crystallised, and hence its three-dimensional structure is unavailable. Herein, we review what is known concerning DehL and tentatively identify the amino acid residues important for catalysis based on a comparative structural and sequence analysis with well-characterised l-specific dehalogenases.

## Background

Halogenated organic compounds contain at least one carbon–halogen bond. More than 3800 different, naturally occurring, halogenated organic compounds are present in huge amounts in the biosphere (Gribble 2003). However, even more have been industrially produced, which is attributable to their diverse use in various industrially related products, e.g., agrochemicals, pharmaceuticals, and solvents (Fetzner and Lingens 1994). These compounds have caused serious environmental pollution owing to their direct toxicity, their potentially toxic breakdown products, and their persistence in the environment.

Interestingly, a number of bacteria use halogenated organic compounds as their sole carbon and energy sources, thereby helping to reverse the effects of environmental halogen-associated pollution. These bacteria produce dehalogenases, enzymes that catalyse the cleavage of carbon–halogen bonds in halogenated organic compounds to produce environmentally benign products. Jensen was the first to discover dehalogenases when he isolated bacteria and fungi that grew on halogenated alkanoic acids (Jensen 1957). Jensen was also the first to assay dehalogenases in a cell-free system, a study that triggered almost all subsequent studies on haloalkanoic acid dehalogenases. To date, many dehalogenases from many different organisms have been studied and certain bacteria produce more than one type of dehalogenase (Table 1). Attention to these bacterial dehalogenases has continually increased owing to their potential application in bioremediation of halogenated organic compounds polluted environment as well as their industrial applications, such as site-directed synthesis of isomers of halogenated organic compounds.

**Table 1:** Known haloacid-dehalogenating bacteria and their dehalogenases

Organism	Substrate for growth	Dehalogenase	Substrate for enzyme	References
*P*. *putida* PP3	d,l-2-Chloropropionate, 2,2-dichloropropionate	DehI/DehII	Monochloroacetate, dichloroacetate, d,l-2-chloropropionate, 2,2-dichloropropionate	Senior et al. (1976), Weightman et al. (1979), Slater et al. (1979) and Weightman et al. (1982)
*Rhizobium* sp. RC1	d,l-2-Chloropropionate, 2,2-dichloropropionate	DehD	Monochloroacetate, monobromoacetate, d-2-chloropropionate	Berry et al. (1979), Leigh et al. (1986), Cairns et al. (1996) and Stringfellow et al. (1997)
		DehE	Monochloroacetate, monobromoacetate, dichloroacetate, dibromoacetate, trichloroacetate, tribromoacetate, d,l-2-chloropropionate, 2,2-dichloropropionate	
		DehL	l-2-Chloropropionate, dichloroacetate, dibromoacetate	
*Moraxella* sp. B	Fluoroacetate	H-I	Fluoroacetate	Kawasaki et al. (1981a, b)
		H-II	Monochloroacetate, monobromoacetate, monoiodoacetate, dichloroacetate, d,l-2-chloropropionate	
*Pseudomonas putida* 109	d,l-2-Chloropropionate	Deh 109	Monochloroacetate, monobromoacetate, monoiodoacetate, l-2-chloropropionate, 2,2-dichloropropionate, d,l-2-bromopropionate d,l-bromobutyrate	Motosugi et al. (1982a)
*Pseudomonas* sp. 113	d,l-2-Chloropropionate	Haloalkanoic acid dehalogenase	Monochloroacetate, monobromoacetate, monoiodoacetate, d,l-2-chloropropionate, d,l-2-bromopropionate 2,2-dichloropropionate, d,l-2-bromo-*n*-butyrate	Motosugi et al. (1982b)
*Pseudomonas* sp. CBS3	2-Chloroacetate, 4-chlorobenzoate	DehCI	Monochloroacetate, monobromoacetate, l-2-chloropropionate, dichloroacetate, 2,2-dichloropropionate,	Klages et al. (1983), Schneider et al. (1991) and Mörsberger et al. (1991)
		DehCII	Monochloroacetate, Monobromoacetate, l-2-chloropropionate	
*Xanthobacter autotrophicus* GJ10	Dichloroacetate, dibromoacetate, d,l-2-chloropropionate, 1,2-dichloroethane	DhlA	Chloromethane, chloroethane, bromoethane, 1,2-dichloroethane, 1,2-dibromoethane, 1-chloropropane, 3-chloropropene, 1-bromopropane, 1,3-dichloropropane, 1-chlorobutane, 1-iodopropane	Janssen et al. (1985), Keuning et al. (1985) and Van der Ploeg et al. (1991)
		DhlB	Monochloroacetate, monobromoacetate, l-2-chloropropionate, dichloroacetate dibromoacetate	
*Burkholderia cepacia* MBA4	Monobromoacetate, monochloroacetate, 2-bromopropionate	DehIVa	Monobromoacetate, monochloroacetate, dichloroacetate, l-2-chloropropionate, l-2-bromopropionate	Tsang et al. (1988)
*Alcaligenes* sp. CC1	2-Chlorobutyrate, 2-chloropropionate, monochloroacetate, *trans*, *cis*-3-chlorocrotonate, 3-Chlorobutyrate	Haloalkanoic acid dehalogenase	Monochloroacetate, 2-chloropropionate, 2,2-dichloropropionate, dichloroacetate	Kohler-Staub and Kohler (1989)
*P*. *putida* strain AJ1	d,l-2-Chloropropionate	HadD	Monochloroacetate, monobromoacetate, d-2-chloropropionate, 2,2-dichloropropionate, 2-bromobutyrate, 2-chloro-2-butyrate	Smith et al. (1990), Jones et al. (1992) and Barth et al. (1992)
		HadL	Monochloroacetate, Monobromoacetate, l-2-chloropropionate, 2,2-dichloropropionate, 2-bromobutyrate, 2-chlorobutyrate	
*Alcaligenes xylosoxidans* ABIV	2,2-Dichloropropionate	DhlC	Monochloroacetate, monobromoacetate, 2,2-dichloropropionate, d,l-2-chloropropionate, 2-chlorobutyrate	Brokamp and Schmidt (1991)
*Ancylobacter aquaticus*	Chloroacetate, d,l-2-chloropropionate, 2-chloroethanol, 1,2-dichloroethane	DhlA	1-Chlopropropane, 1-chlorobutane, 1,2-dichloroethane, 1,2-dibromopropane, 1,3-dichloropropane, 1,4-dichlorobutane	Van den Wijngaard et al. (1992)
*Pseudomonas fluorescens* 1	Fluoroacetate	Haloalkanoic acid dehalogenase	Not determined	Wong et al. (1992)
*Pseudomonas acidovoran*				
*Pseudomonas* sp. YL	d,l-2-Chloropropionate	d,l-DEX	Monochloroacetate, monobromoacetate, monoiodoacetate, d,l-2-chloropropionate, d,l-2-chloro-*n*-butyrate	Liu et al. (1994)
		l-DEX	Monochloroacetate, monobromoacetate, monoiodoacetate, l-2-chloropropionate, 2,2-dichloropropionate, d,l-2-chloro-*n*-butyrate	
*Burkholderia* sp. FA1	Fluoroacetate	FAc-DEX FA1	Monofluoroacetate, monochloroacetate, monobromoacetate	Kurihara et al. (2003)
*Bradyrhizobium* sp.	2,2-Dichloropropionate	Haloalkanoic acid dehalogenase	Not determined	Marchesi and Weightman (2003)
*Rhodococcus* sp.	3-Chloropropionate	Haloalkanoic acid dehalogenase	3-Chlorolactate, 3-chloropropionate, 3-chlorobutyrate, 2,3-dichloropropionate, 2,2,3-trichlorobutyrate	Jing and Huyop (2007a)
	3-Chlorobutyrate			
*Methylobacterium* sp. HN2006B	2,2-Dichloropropionate	Haloalkanoic acid dehalogenase	Not determined	Jing and Huyop (2007b)
*Pseudomonas* sp. R1	Monochloroacetate	Haloalkanoic acid dehalogenase	Not determined	Ismail et al. (2008)
*Methylobacterium* sp. HJ1	2,2-Dichloropropionate	Haloalkanoic acid dehalogenase	Dichloroacetate, 2-chloropropionate, 2,2-dichloropropionate, 2,2-dichlobutyrate	Jing and Huyop (2008)
*Pseudomonas* sp. B6P	3-Chloropropionate	Haloalkanoic acid dehalogenase	3-Chloropropionate, 2,3-dichloropropionate	Mesri et al. (2009)
*P*. *putida* S3	d,l-2-Chloropropionate	DehD/DehL	Monobromoacetate, monoiodoacetate, monochloroacetate, d-2-chloropropionate, l-2-chloropropionate	Thasif et al. (2009)
*Bacillus* sp. TW1	Monochloroacetate	Haloalkanoic acid dehalogenase	Not determined	Zulkifly et al. (2010)
*Aminobacter* sp. SA1	2,2-Dichloropropionate, d,l-2-chloropropionate	Haloalkanoic acid dehalogenase	Not determined	Amini et al. (2011)
*Bacillus megaterium* GS1	2,2-Dichloropropionate	Haloalkanoic acid dehalogenase	Not determined	Roslan et al. (2011)
*Labry*s sp. Wy1	2,2-Dichloropropionate	Haloalkanoic acid dehalogenase	Not determined	Wong and Huyop (2011)
*Serratia marcescens* sp. SE1	2,2-Dichloropropionate	Haloalkanoic acid dehalogenase	Not determined	Abel et al. (2012a)
*Ralstonia solancearum* strain 121002, *Acinobacter baumannii* strain 121007, *Chromobacterium violaceum* strain 121009	2,2-Dichloropropionate	Haloalkanoic acid dehalogenase	2,2-Dichloropropionate, d,l-2-chloropropionate	Abel et al. (2012b)
*Ancylobacter dichloromethanicus*	Fluoroacetate	Haloalkanoic acid dehalogenase	Not determined	Camboim et al. (2012a)
*Pigmentiphaga kullae*				
*Paenibacillus* sp.	Fluoroacetate	Haloalkanoic acid dehalogenase	Not determined	Camboim et al. (2012b)
*Cupriavidus* sp.				
*Ancylobacter* sp.				
*Ralstonia* sp.				
*Stenotrophomonas* sp.				
*Staphylococcus* sp.				
*Burkholderia* sp. DW	2,2-Dichloropropionate	Haloalkanoic acid dehalogenase	Not determined	Wong and Huyop (2012)
*Enterobacter cloacea* D9				
*Arthrobacter* sp. S1	2,2-Dichloropropionate, d,l-2-chloropropionate, 3-chloropropionate	Haloalkanoic acid dehalogenase	Not determined	Bagherbaigi et al. (2013)
*Arthrobacter* sp. strain D2	Monobromoacetate, 2,2-dichloropropionate, d,l-2-chloropropionate	Haloalkanoic acid dehalogenase	Not determined	Alomar et al. (2014)
*Arthrobacter* sp. strain D3	Monochloroacetate	Haloalkanoic acid dehalogenase	Not determined	Alomar et al. (2014)
*Labrys* sp. strain D1				
*Bacillus* sp. strain EK1	2,2-Dichloropropionate, 2,3-dichloropropionate, d,l-2-chloropropionate	Haloalkanoic acid dehalogenase	Not determined	Khosrowabadi and Huyop (2014)
*Rhodococcus* sp. strain EK2				
*Lysinibacillus* sp. EK3				
*Microbacterium* sp. strain EK4				
*Aminobacter* sp. strain EK5				
*Raoutella ornithilolytica*	2,2-Dichloropropionate	Haloalkanoic acid dehalogenase	Not determined	Niknam et al. (2014)

The fast growing, soil *Rhizobium* sp. RC1 uses 2,2-dichloropropionate, d,l-2-chloropropionate, and d,l-2-bromopropionate as its sole carbon and energy sources (Allison et al. 1983). The organism produces three different dehalogenases, d-2-haloacid dehalogenase (DehD), l-2-haloacid dehalogenase (DehL), and the dual isomeric haloacid dehalogenase (DehE) (Leigh et al. 1986). Herein, we focus mainly on these haloacid dehalogenases, with special emphasis on *Rhizobium* sp. RC1 DehL, and propose, based on an amino acid sequence alignment and structural comparison, the DehL residues that are likely involved in catalysis.

### Classification of haloacid dehalogenases

Generally, haloacid dehalogenases are classified according to their substrate specificities and the configuration of their products. Given these criteria, Slater and colleagues classified haloacid dehalogenases as Class 1L that acts specifically on l-2-haloalkanoic acids to produce the corresponding d-2-hydroxyalkanoic acids; Class 1D that acts specifically on d-2-haloalkanoic acids to produce l-2-hydroxyalkanoic acids; Class 2I (inversion-type dehalogenase) that dehalogenates d- and l-2-haloalkanoic acids to produce the corresponding 2-hydroxyalkanoic acids with inverted configurations; and Class 2R (retention-type dehalogenase) that dehalogenates both isomers of 2-haloalkanoic acids to produce the corresponding 2-hydroxyalkanoic acids that have the same configurations as their substrates (Slater et al. 1997).

Notably, investigating the evolutionary relationships between dehalogenases using substrate specificities as the only criterion can be misleading. For example, *Rhizobium* sp. RC1 DehL acts only on l-2-chloropropionate, yet its gene sequence (Cairns et al. 1996) differs substantially from the sequences of other bacterial dehalogenases with the same substrate specificity. Therefore, *Rhizobium* sp. RC1 DehL was tentatively suggested to be the first member of a new group (Hill et al. 1999). In addition, based on an alignment of translated amino acid sequences, *Moraxella* sp. *dehH1* encoding fluoroacetate dehalogenase H-I (Kawasaki et al. 1981a, b), was proposed to be related to the haloalkane dehalogenase genes *dhlA* from *Xanthobacterautotrophicus* (Keuning et al. 1985) and *dhaA* from *Rhodococcus rhodochrous* (Curragh et al. 1994); and the α-hexachlorocyclohexadiene dehalogenase gene *linB* from *Pseudomonaspaucimobilis* (Nagata et al. 1993), suggesting the existence of an addition group of haloalkanoic acid dehalogenases (Hill et al. 1999).

In an effort to establish a robust molecular phylogenetic classification and to strengthen framework for studies of bacterial dehalogenases; Hill and colleagues designed degenerate PCR primer pairs for specific amplification and isolation of group I and II dehalogenases (Hill et al. 1999). The dehalogenases in these two distinct groups have fundamentally different mechanisms, indicating that they are not evolutionarily related. Group II are stereo-selective, dehalogenating l- but not d-2-chloropropionate while group I comprises non-stereo-selective and d-2-chloropropionate specific dehalogenases. Because the classification system based on Hill and colleagues’ degenerate PCR primer pairs uses molecular genetic information, it provides a more robust and convincing set of dehalogenase classes, which has led it to be widely adopted.

All the aforementioned dehalogenase classes contain well-studied dehalogenases that target one or more halogen atoms at the α-carbon (i.e. C2) position. In addition, dehalogenases that act on β(3)-halo-substituted alkanoic acids also exist. In 1979, Slater and colleagues showed that a crude extract from *Pseudomonas putida* PP3 contained a 2-chloropropionate dehalogenating activity and a small amount of activity against 3-chloropropionate. Recently, these dehalogenases have received more attention with many studies reporting on dehalogenases that remove chloride from the β-carbon of chloroalkanoic acids (Jing and Huyop 2007a; Yusn and Huyop 2009; Mesri et al. 2009).

### *Rhizobium* sp. RC1 haloacid dehalogenases

**Fig. 1:**
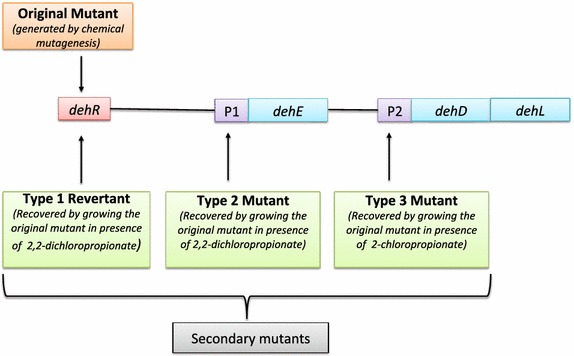
Proposed genetic organisation and regulation for the *Rhizobium* sp. RCI dehalogenase genes. R represents regulator gene that controls all three dehalogenases. P1 and P2 represent promoter regions of the structural genes, *dehE*, and *dehD*/*dehL* respectively. The *arrows* indicate sites of mutations. Original mutant lack the ability to express any of the three dehalogenase structural genes. Type 1 revertant regained the wild type ability to express all the three dehalogenase genes. Type 2 and 3 are constitutive for DehE and DehD/DehL respectively

In 1979, Berry and colleagues isolated a fast-growing soil bacterium capable of using 2,2-dichloropionate as its sole carbon and energy sources, which they tentatively identified as *Rhizobium* sp. RC1 (Berry et al. 1979). The bacterium was reported to express three dehalogenases induced by different haloalkanoic acids (Allison et al. 1983). These dehalogenases were genetically characterized using a series of mutant strains (Leigh et al. 1986). The *Rhizobium* sp. RC1 mutant strain produced by chemical mutagenesis cannot use 2,2-dichloropropionate or d,l-2-chloropropionate as its sole carbon and energy sources. Three secondary mutants were isolated after culturing the original mutant strain on 2,2-dichloropropionate and/or d,l-2-chloropropionate-containing agar. In the presence of 2,2-dichloropropionate two secondary mutants, types 1 and 2 were recovered. The type 1 reverted to the wild-type phenotype (revertant), for which all three dehalogenase activities could be induced. The type 2 mutant constitutively produced DehE, but DehL and DehD were not expressed under any of the tested conditions. The selective pressure induced by the presence of d,l-2-chloropropionate resulted in the type 3 mutant that constitutively produced DehL and DehD but could not produce DehE. The mutation sites in the original mutant strain have not been identified, however they were proposed to be within the regulator gene (Leigh et al. 1986), which would affect production of the three dehalogenases provided that their genes are all controlled by this regulator. Obtaining the type 1 revertant (wild-type phenotype) requires a reversion of the original mutation in the regulator gene, or a repressor mutation in the regulator gene. Similarly to produce the type 2 and 3 secondary mutants, separate mutations in the promoter regions controlling expression of DehE and DehD/DehL are required respectively (Fig. 1).

The stereospecificities of the three *Rhizobium* sp. RC1 dehalogenases were characterised further by Huyop and Cooper (2003) and Huyop et al. (2004). DehL degrades l-2-chloropropionate and dichloroacetate; DehD is active against d-2-chloropropionate and monochloroacetate; and DehE dehalogenates 2,2-dichloropropionate, d,l-2-chloropropionate, monochloroacetate, dichloroacetate, and trichloropropionate. The lactates produced from d- or l-2-chloropropionate by the three dehalogenases have inverted configurations (Leigh et al. 1988). All these three dehalogenases can also act on 2,3-dichloropropionate with 2-hydroxy-3-chloropropionate being the assumed product. DehE can act on brominated substrates and does so more rapidly than it does to chlorinated substrates (Huyop et al. 2004). Huyop and colleagues assessed the specificity of DehE against mono-, di-, and tri-chloroacetates and the corresponding bromoacetates. All tested bromoacetates had greater associated specificity constants (a determinant of catalytic efficiency) than did their corresponding chloroacetates, suggesting that the brominated compounds would be the preferred DehE substrates (Huyop et al. 2004). DehL and DehD also use dibromoacetate and monobromoacetate, respectively, as substrates, although they are more active against the corresponding chlorinated substrates (Huyop and Cooper 2003). *Rhizobium* sp. RC1 DehD is the most kinetically active d-specific dehalogenase found (Huyop and Cooper 2003), suggesting that it would be the best d-specific dehalogenase for industrial production of l-specific products. For example, in the industrial production of herbicides and pharmaceuticals, DehD can be used instead of the d-2-haloacid dehalogenase from *Pseudomonas* in conjunction with a chiral feedback chemical to produce the l-2-chloropropionate intermediate (Taylor 1990).

### *Rhizobium* sp. RC1 dehalogenase genes and their regulation

The *Rhizobium* sp. RC1 genes encoding the three dehalogenases have been sequenced, and the location of *dehD* is 177 non-coding base pairs upstream of *dehL* (Fig. 1). Conversely, the location of *dehE* relative to that of the other two is not known (Cairns et al. 1996). The deduced DehL and DehD amino acid sequences are only 18 % identical, indicating that these dehalogenases probably do not have many common features (Cairns et al. 1996). This degree of sequence identity is similar to that found for *P*. *putida* AJ1 HadD and HadL (Barth et al. 1992; Jones et al. 1992).

The deduced amino acid sequence of DehE is not significantly similar to those of DehD and DehL, suggesting no obvious evolutionary linkage between *dehE* and *dehD* or *dehL* (Stringfellow et al. 1997). By characterising the expression of mutant strains of *Rhizobium* sp. carrying one or more mutations in *dehE*, *dehD*/*dehL* or *dehR* genes, it was found out that the *dehR* encoding for a regulatory protein (DehR) probably controls the three dehalogenase structural genes. The proposed regulatory model involves DehR binding to and activating the promoter of the dehalogenase structural genes thereby allowing for their transcription. However, this binding only occurs in the presence of d,l-2-chloropropionate and/or 2,2-dichloropropionate as the inducers (Leigh et al. 1986). *dehR* has been located upstream of *deh*E and its product, DehR was proved to control *dehE* in an engineered *E*. *coli* expression system (Huyop and Cooper 2014).

Notably, expression of cloned *dehD* and *dehL* is dependent on the presence of a co-transformed *lac* promoter upstream of these genes in a *dehD*- and *dehL*-containing plasmid (Cairns et al. 1996), indicating that the regulatory sequence was not cloned or that it was not functional in the *E*. *coli* host. Therefore, a single promoter possibly regulates *dehD* and *dehL* expression and physically differs from that regulating *dehE*. However, the regulatory mechanism(s) for these genes is not fully understood. Additional studies are needed to provide a clearer picture of how these genes are regulated.

### Relationships between *Rhizobium* sp. RC1 dehalogenase sequence and activities

The amino acid sequence of *Rhizobium* sp. RC1 DehE is similar to that of *P*. *putida* PP3 DehI, suggesting that the two enzymes have similar structures, functions, and the same catalytic residues (Hamid et al. 2011). A structural model of DehE was built using DehI as the template (Hamid et al. 2013). The involvement of various amino acid residues at the presumed DehE catalytic active site was assessed by site-directed mutagenesis, which identified TYR34, PHE37, SER188, and ASP189 as catalytically important (Hamid et al. 2015b). DehE is inactive against β-haloalkanoic acids, e.g., 3-chloropropionate. However, when SER188 was mutated to VAL it gained activity against 3-chloropropionate (Hamid et al. 2015a).

In DehD, ARG134, ARG16, and TYR135 are proposed to be necessary for catalysis, with ARG134 playing the key role in stereospecific substrate binding (Sudi et al. 2014a, b). Currently, 3D structure information concerning DehL is unavailable. New studies to determine the catalytic and substrate-interacting residues of DehL, and a three-dimensional structure for it are needed to gain insight into its reaction mechanism and to maximise its industrial and environmental benefits.

### l-Stereospecific dehalogenases

#### Diversity of l-stereospecific dehalogenases

Many organisms produce l-stereospecific dehalogenases probably because most naturally occurring halogen-containing organic compounds exist in the l-form (Martínez-Rodríguez et al. 2010). Some of the genes encoding these enzymes have been sequenced (Table 2). Although, most known dehalogenases are proteobacterial in origin, Gram-positive bacteria, e.g., *Rhodococcus*, also degrade haloacid compounds (Jing and Huyop 2007a, b). The Gram-positive bacterium, *Staphylococcus* sp. produces a haloalkanoic dehalogenase (Camboim et al. 2012a) when this microbe is present in the soil of fluoroacetate-producing plants, a selective-pressure condition. Also, the thermophilic bacterium *Sulfolobus tokodaii* strain 7, isolated from the Beppu spring in Kyushu Japan in 1983, contains the l-stereospecific dehalogenase L-HAD. This acidophilic bacterium grows optimally at 80 °C, and its genome has been fully sequenced (Kawarabayasi et al. 2001), which is how L-HAD was initially identified. Characterisation of this dehalogenase suggested that it is maximally active at 60 °C (Rye et al. 2009). L-HAD tolerates pH environments between 4 and 10, and remains fully active after incubation at 70 °C for 4 h (Bachas-Daunert et al. 2009).

**Table 2:** l-2-Haloacid dehalogenases from different organisms

Dehalogenase	Organism	NCBI accession no.	References
DehL	*Rhizobium* sp. RC1	CAA63794.1	Cairns et al. (1996)
HadL	*P*. *putida* AJ1	M81841.1	Barth et al. (1992) and Jones et al. (1992)
DhlB	*X. autotrophicus* GJ10	M81691.1	van der Ploeg et al. (1991)
DehH109	*P*. *putida* 109	D17523.1	Kawasaki et al. (1994)
HehIVa	*B. cepacia* MBA4	X66249.1	Murdiyatmo et al. (1992)
H-II	*Moraxella* sp. B	D90423.1	Kawasaki et al. (1992)
L-DEX	*Pseudomonas* sp. YL	S74078.1	Nardi-Dei et al. (1994)
DehII	*P*. *putida* PP3	AJ133462.1	Hill et al. (1999)
DehCI	*Pseudomonas* sp. CBS3	M62908.1	Schneider et al. (1991)
DehCII	*Pseudomonas* sp. CBS3	M62909.1	Schneider et al. (1991)
L-HAD	*Sulfolobus tokodaii* 7	NC_003106.2	Kawarabayasi et al. (2001)

**Fig. 2:**
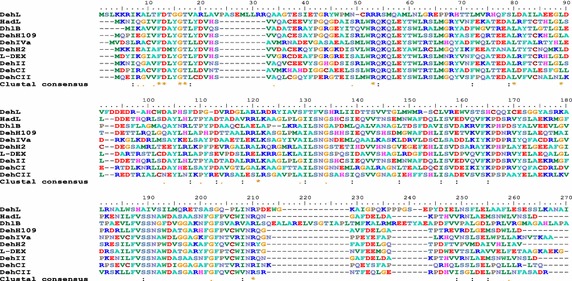
Multiple sequence alignment of l-2-dehalogenases by ClustalW2 (Larkin et al. 2007). The percentage of sequence identity for DehL and the following dehalogenases: 5 %, HadL; 16 %, DehCII; 15 %, DhlB; 16 %, DehH109; 13 %, DehIVa; 15 %, DehH2; 17 %, l-DEX; 16 %, DehII; and 14 %, DehCI

Interestingly, even though the l-2-haloalkanoic dehalogenases specifically target l-isomers of haloacids, their gene and the deduced amino acid sequences are not all similar. The sequences of *Rhizobium* sp. RC1 DehL and other l-2-haloalkanoic dehalogenases have <18 % sequence identity (Fig. 2). Conversely, substantial sequence similarities are found for non-DehL l-2-dehalogenases (sequence identities from 33 to 96 %). Notably, *P*. *putida* AJ1 HadL and *P*. *putida* PP3 DehII have almost identical amino acid sequences (~96 % identity).

#### The residues catalytically important in l-specific dehalogenases

Mutation of certain residues in l-2-dehalogenases significantly affects their catalytic abilities. The catalytically important residues are highly conserved in l-2-dehalogenases. These residues are well characterised in *Pseudomonas* sp. YL, l-DEX (Kurihara et al. 1995). Its crystal structure (Hisano et al. 1996a, b) and those of *X*. *autotrophicus* GJ10 DhlB (Ridder et al. 1995, 1997), *Burkholderia cepacia* MBA4 DehIVa (Schmidberger et al. 2007) and *S*. *tokodaii* 7 L-HAD (Rye et al. 2007) have been solved. Kurihara and colleagues identified the catalytically important residues in *Pseudomonas* sp. YL l-DEX by site-directed mutagenesis (Kurihara et al. 1995) that involved replacing its highly conserved charged and polar residues (except for the N-terminal Met) with other residues. The genes encoding the mutated proteins were expressed in large amounts under appropriate conditions, purified, and tested for activity towards l**-**2-chloropropionic acid. The replacement of ASP10, ASP180, ARG41, LYS151, SER175, SER118, THR14, TYR157, and ASN177 caused significant activity decreases. Because replacement of these residues did not cause conformational changes detectable by spectrophotometry and gel filtration, these residues are probably essential for catalysis. ASP10 was suggested to be the catalytic nucleophile (Liu et al. 1995); however, its replacement with ASN did not completely inactivate the enzyme, whereas replacement with ALA, GLY, or GLU did completely inactivate the enzyme (Kurihara et al. 1995). Possibly ASN10 was deamidated, resulting in the wild-type Asp, or it served as a weaker, but still active nucleophile (Ichiyama et al. 2000; Kurihara and Esaki 2008).

The residues found to be essential in l-DEX, are conserved in DhlB and DehIVa from *X*. *autotrophicus* GJ10 and *B*. *cepacia* MBA4, respectively. The crystal structure analyses of reaction intermediates of DhlB (Ridder et al. 1997) and DehIVa (Schmidberger et al. 2007) suggest functional conservation among the conserved catalytically important residues. However, no site-directed mutagenesis studies have been performed to confirm this supposition.

#### Generalised catalytic mechanisms for l-2-haloacid dehalogenases

The most extensively studied l-2-haloacid dehalogenases are *Pseudomonas* sp. YL l-DEX and *X*. *autotrophicus* GJ10 DhlB. Both have been crystallised, and their dehalogenation mechanisms are well understood (Hisano et al. 1996a, b; Ridder et al. 1995). Given this information, it has been proposed that l-2-haloacid dehalogenases catalyse the hydrolytic cleavage of carbon–halogen bonds (Eq. 1) by similar mechanisms (Hisano et al. 1996b).

1 $$\begin{aligned} & RCHXCOOH + OH^{ - } \to RCHOHOOH + X^{ - } \\ & R = {\text{H or alkyl group}},\quad X = {\text{ halogen atom}} \\ \end{aligned}$$

**Fig. 3:**
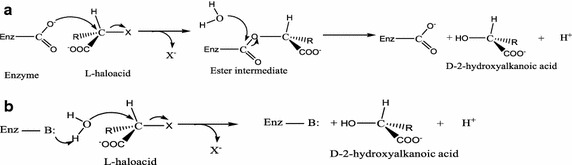
Proposed catalytic reaction mechanisms for l-2-haloacids dehalogenases. **a** Attack on the C2 of the substrate by a dehalogenase side-chain carboxyl to produce an ester intermediate with subsequent attack by a water molecule on the intermediate to produce the corresponding hydroxyacid with the opposite stereo-configuration. **b** Water is activated by a basic residue and attacks the substrate to produce the hydroxyacid with simultaneous release of halide ion. Adapted from Kurihara et al. (1995)

At the atomic level, the release of a halide by an l-2-haloacid dehalogenase probably proceeds by an S_N_2 reaction, during which the halide is replaced by a hydroxyl by one of two possible mechanisms (Fig. 3), which is based on Figure 2 in Kurihara et al. (1995). One possible reaction involves an initial nucleophilic attack on the C2 of the substrate on the side opposite that of the halide by a side-chain carboxyl of an acidic dehalogenase residue. All moieties attached to the C2 atom except the halogen are planer in the transition state, such that the nucleophilic carboxyl oxygen interacts with the C2 atom perpendicular to the plane of the transition state and inversion of the C2 stereochemistry occurs with release of the halide. Subsequently, an activated catalytic water molecule cleaves the intermediate, with retention of the C2 stereochemistry, releasing the 2-hydroxyl acid product and the intact enzyme (Kurihara et al. 1995; Li et al. 1998). As noted above, ASP10 was suggested to be the nucleophile in the dehalogenases from *Pseudomonas* sp. YL (Liu et al. 1995) *X*. *autotrophicus* GJ10 (Ridder et al. 1997) and *B*. *cepacia* MBA4 DehIVa (Schmidberger et al. 2007).

**Fig. 4:**
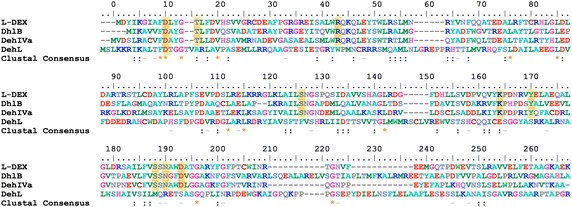
Multiple sequence alignment of DehL, l-DEX, DhlB, and DehIVa. The *shaded positions* indicate the residues important to l-DEX, DhlB, and DehIVa catalysis. Sequence numbers are those of l-DEX

A second possible mechanism involves a water molecule, activated by a basic residue, attacking the substrate to release the halide thereby producing the 2-hydroxyl acid product in a single step (Kurihara et al. 1995).

### Possible catalytically important residues in *Rhizobium* sp. RC1 DehL

**Fig. 5:**
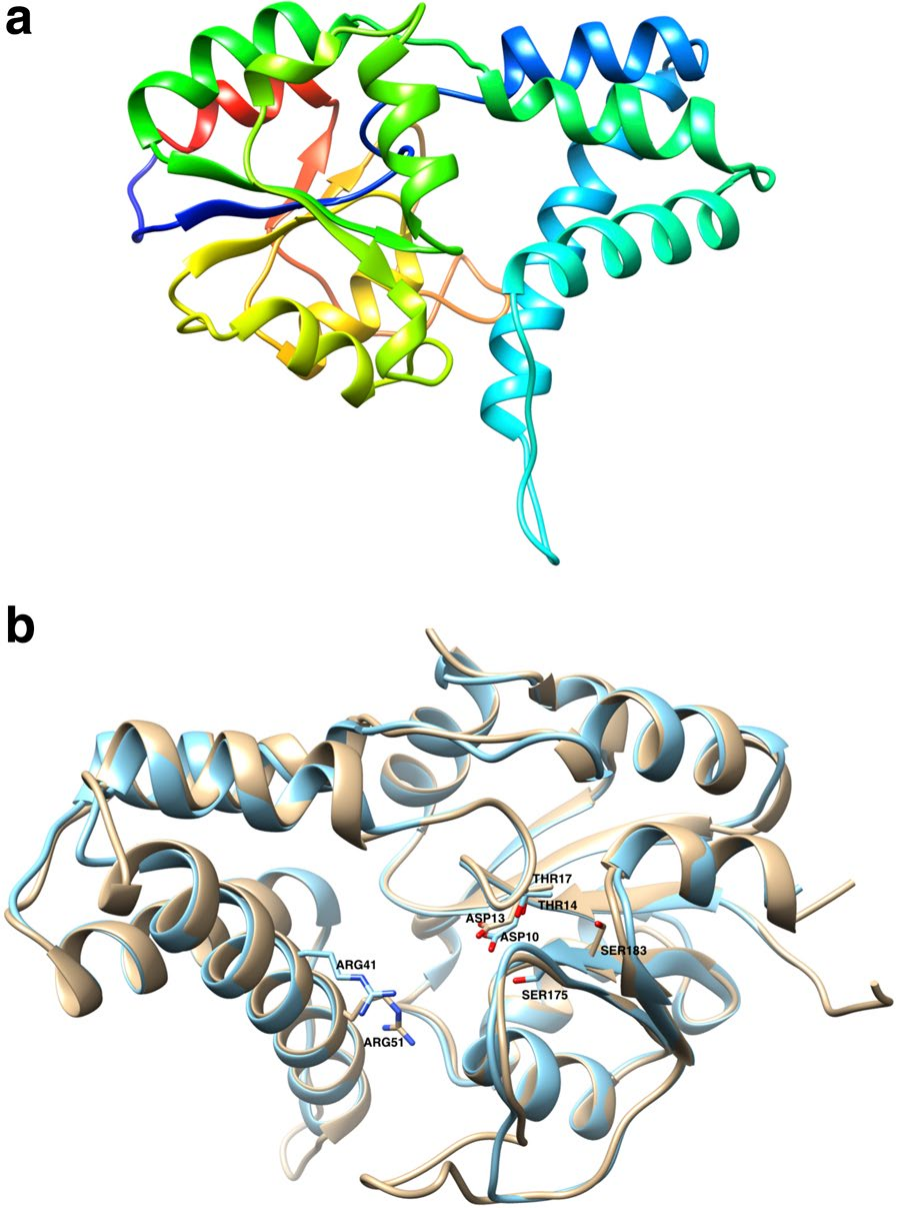
3D homology model of DehL **a** 3D structure of DehL in cartoon representation. The structure is in reverse *rainbow colour* sequence with amino terminal in *violet* and carboxylic terminal in *red*. **b** Superposition of DehL (in *brown*) and l-DEX structure (in *blue*). The side chains of the conserved catalytically important residues (ASP13, THR17, ARG51 and SER183 in DehL corresponding to ASP10, THR14, ARG41 and SER175) are shown in stick representation

The crystal structure of *Rhizobium* sp. RC1 DehL is not available. Nor has any study directly identified the residues involved in DehL catalysis. Even though no obvious sequence identity between DehL and other l-2-haloacid dehalogenases exists, 3D structure comparison of DehL and l-DEX; and multiple alignment of the DehL sequence with those of l-DEX, DhlB, and DehIVa allow us to infer the possibly catalytically important DehL residues. 3D structure of DehL (Fig. 5a) was predicted by Modeller 9.15 using l-DEX (PDB IB: 1JUD) as template. Structural superposition of DehL with l-DEX (Fig. 5b) and multiple sequence alignment of DehL, l-DEX, DhlB, and DehIVa (Fig. 4; Table 3) show ASP10, THR14, ARG41 and SER175, which have been shown to affect catalytic activity in l-DEX are conserved in DehL. However, THR14, ARG41 and SER175 were observed to be only structurally but not sequentially conserved in DehL. This is probably due to variation in size of the aligned sequences. The ARG51 of DehL is not in directly similar structural position as the ARG41 of l-DEX, although the positions of the two ARG residues are in relative position and pointing at the same direction in the active site. The variation in positions of the ARG residues in the two dehalogenases might be due to the difference in size of the active site, which is dependent on the range of substrate specificities. For example l-DEX activity is not limited to short-carbon-chain 2-haloacids such as monochloroacetate but it also acts on long-carbon-chain of 2-haloacids such as 2-bromohexadecanoate in *n*-heptane (Liu et al. 1994); whereas l-2-chloropropionate is the longest carbon-chain DehL ever reported to acts on.

**Table 3:** Residues important for catalysis in the crystallised dehalogenases and predicted for *Rhizobium* sp. RC1 DehL

Key amino acid residues			Predicted *Rhizobium* sp. RC1 dehalogenase residues important for catalysis
l-DEX	DhlB	DehIVa	DehL
D10	D8	D11	D13
T14	T12	T15	T17
R41	R39	R42	R51
S118	S114	S119	–
K151	K147	K152	–
Y157	Y135	Y158	–
S175	S171	S176	S183
N177	N173	N178	–
D180	D176	D181	–

Conservation in amino acid often confers functional conservation. Therefore, it can be hypothesise that the catalytically important residues of l-DEX that are conserved in DehL may also be catalytically important and probably have similar functions. This was reported to be the case among the nine conserved catalytically important residues in l-DEX, DhlB and DehIVa. ASP10 in l-DEX that corresponds to ASP13 in DehL plays a nucleophilic role by attacking C2 of L-2-chloropropionate during dehalogenation catalysis (Liu et al. 1995). The corresponding residues in DhlB (ASP8) (Ridder et al. 1997) and DehIVa (ASP11) (Schmidberger et al. 2007) were reported to have similar function. SER 175 in l-DEX (SER183 in DehL) and its corresponding residue, SER171 in DehIVa both involve in a hydrogen bond with ASP10 to probably maintain the orientation of its carboxyl group in a way suitable to attack the C2 of the substrate (Hisano et al. 1996a, b; Schmidberger et al. 2007). As a positively charged polar residue, ARG41 in l-DEX (ARG 51in DehL) accepts the released chloride ion by electrostatic interaction (Kondo et al. 2014). Furthermore, the corresponding residue in DehIVa (ARG42) was proposed to play key role in substrate “lock down” mechanism; and also acts a member of the halide-binding cradle together with ASN120 and TRP180 (Schmidberger et al. 2007). The role of THR14 in l-DEX (THR17 in DehL) is not yet determined, however its corresponding residue in DhlB (THR12) together with SER171 and ASN173 were reported to firmly hold the ASP8 in a position that favours the nucleophilic attack (Ridder et al. 1997). On the other hand, the rest of the catalytically important residues of l-DEX (SER118, LYS 151, TYR157, ASN177 and ASP180) not conserved in DehL may be probably the same as those in l-DEX but in different positions in the active site or substituted by similar residues. To fully elucidate the mechanism of DehL dehalogenation and the contributions of the specific residues, additional work is needed.

## Conclusions

l-2-Haloacid dehalogenases have been found in many different bacteria; many of these enzymes have been sequenced, and for some, their substrate specificities and kinetics have been well characterized. In addition, four have been crystallised and their three-dimensional structures solved, which is informative concerning their possible catalytic mechanism(s). Although, DehL from *Rhizobium* sp. RC1 dehalogenates the same substrates as l-2-haloacid dehalogenases from other organisms do, its amino acid sequence is quite different from those of the other enzymes. Results of our pairwise DehL amino acid sequence comparison with those of the crystallised proteins; and the structural superposition of DehL and l-DEX suggest that ASP10, THR14, ARG 41 and SER 175 are conserved in DehL and the corresponding residues may be catalytically important in DehL dehalogenation reaction.
